# Differences in microorganism profile in periprosthetic joint infections of the hip in patients affected by chronic kidney disease

**DOI:** 10.1186/s10195-024-00806-x

**Published:** 2024-12-19

**Authors:** Davide Stimolo, Maximilian Budin, Domenico De Mauro, Eduardo Suero, Thorsten Gehrke, Mustafa Citak

**Affiliations:** 1 Helios ENDO-Klinik, Holstenstrasse 2, 22767 Hamburg, Germany; 2 Musculoskeletal Oncology Unit, Department of Orthopedics, University of Florence, Largo Palagi 1, 50135 Florence, Italy; 3 Department of Public Health, Orthopedic Unit, “Federico II” University, Naples, Italy; 4 Department of Orthopedics and Geriatric Sciences, Catholic University of the Sacred Heart, Largo F. Vito 8, 00168 Rome, Italy; 5 Second Department, Orthopaedic Hospital Vienna-Speising, Speisinger Straße 109, 1130 Vienna, Austria; 6 Department of Orthopaedics and Trauma Surgery, Musculoskeletal University Center Munich (MUM), University Hospital, LMU Munich, Marchioninstrasse 15, 81377 Munich, Germany

**Keywords:** Periprosthetic Joint infection, Bacteria, Microorganism profile, Chronic kidney disease, Chronic kidney failure, Total hip arthroplasty

## Abstract

**Background:**

Patients affected by chronic kidney disease (CKD) are at increased risk of periprosthetic joint infection (PJI) after total hip arthroplasty (THA). This patient population has a higher risk of recurrent infections and hospitalization. The aim of this study is to compare the profile of microorganisms in patients with CKD and PJI of the hip versus controls and to individuate potentially unusual and drug-resistant microorganisms among the causative bacteria.

**Materials and methods:**

A total of 4261 patients affected by PJI of the hip were retrospectively studied. Patients affected by CKD in this population were identified and compared with a control group of patients with PJI but without CKD. Data on patient characteristics and comorbidities were collected. The microorganisms responsible for PJI were identified and compared between both groups.

**Results:**

The CKD group included 409 patients, 54.3% male, mean age of 73.8 ± 8.9 years, a higher body mass index (BMI) than the general population (29.88 ± 5.90 kg/m^2^), and higher age-adjusted CCI of 6.15 ± 2.35. Overall, 70 different isolates of microorganisms were identified, including 52 Gram-positive spp., 28 Gram-negative spp., 3 fungi, and 1 mycobacterium. Polymicrobial infections were more common in CKD group than controls (47.9% versus 30.9%; *p* < 0.0001). *Staphylococcus* spp. were the most common bacteria in both groups, followed by Gram-negative Enterobacteriaceae and *Streptococcus* spp. CKD group showed a higher risk of developing infections caused by *Staphylococcus aureus* (*p* = 0.003), Gram-negative bacteria, and *Candida* (*p* = 0.035).

**Conclusions:**

Renal failure exposes patients who undergo THA to PJI caused by microorganisms that are potentially more drug resistant, leading to a higher risk of treatment failure. Knowing in advance the different microorganism profiles could help to plan a different surgical strategy.

*Level of Evidence* III.

## Introduction

Chronic kidney disease (CKD) affects 8–16% of the population worldwide [1]. Around 30% of patients with end-stage hip and knee arthritis requiring total joint replacement (TJR) have chronic renal disease [2]. The risk increases with the severity of CKD. The reported incidence of THA in dialysis-dependent patients is 35 episodes per 10,000 person-years, compared with 5.3 episodes for the general population (RR 6.6) [3]. In patients after renal transplant, avascular necrosis of the femoral head (AVN) is a recognized complication of chronic steroid use and the prevalence of degenerative changes secondary to AVN varies between 5% and 40% [4]. Bone quality in patients with CKD is often poorer than that of other patients owing to renal osteodystrophy and alterations of calcium metabolism. Patients affected by CKD have high risk of perioperative mortality and complications after THA [5]. In a large database study, the odds ratio for mortality and major complications was 3.17 and 1.28, respectively, in patients with CKD compared with controls [6]. In joint replacement surgery, periprosthetic joint infection (PJI) is one of the most feared complications. PJI is associated with multiple reoperations, longer hospital stay, prolonged antibiotic therapies, and high costs for the healthcare system [7]. While the risk of PJI in the general population is 1–3% [8, 9], the risk of PJI in patients with CKD is 2–10% and increases with the severity of renal failure [10]. Patients with CKD have a high risk of hospitalization and of intensive care unit admission [11]. One of the most important causes of hospitalization is sepsis and systemic infectious complications including pneumonia, urinary tract infections (UTIs), and septic shock [2].

In the current study, we aimed to determine whether the complex characteristics of patients with CKD, including a high risk of bacteremia, repeated hospitalizations, and prolonged and repeated antibiotic use, could result in a different causative pathogen profile in patients with CKD affected by PJI after THA. The aim of the study was to identify the most common microorganisms involved in PJI after total hip replacement in patients with CKD and to identify any epidemiological differences compared with the general population.

## Materials and methods

### Study design

A retrospective case–control study was conducted at our tertiary high-volume single-center institution, specialized in joint reconstruction surgery. Data include patients treated in our institution in the period 2008–2020. The study adhered to the principles outlined in the Declaration of Helsinki. The study was conducted with approval from our local Ethics Committee (registration code 300,431-WF).

### Patient characteristics and data extraction

Patients were identified through a query of our institution's electronic health records system. Inclusion criteria were as follows: (i) chronic periprosthetic joint infection of the hip, (ii) patients affected by CKD at diagnosis of PJI. Subjects with incomplete data, culture-negative PJI, with less than 2 weeks of antibiotics interruption before samples were taken were excluded. The diagnosis of PJI followed the principles established at the International Consensus Meeting (ICM) 2018 for periprosthetic joint infections [12]. PJI was defined as chronic if it occurred after 90 days from the index procedure or when symptoms of infection developed for more than 4 weeks [12]. The diagnosis of CKD was defined by a glomerular filtration rate (GFR) of less than 60 mL/min/1.73 m^2^ according to the Kidney Disease: Improving Global Outcomes (KDIGO) 2012 clinical practice guidelines [13, 14]. Patients affected by PJI after THA but free from CKD who were treated at our institution during the same time frame served as the control group.

The following data were assessed for both groups: age, gender, body mass index (BMI), Charlson Comorbidity Index (CCI) [15], clinical history data including comorbidities and chronic diseases, and results of microbiological cultures conducted with appropriate growth media. Cultures were obtained preoperatively through x-ray-guided hip aspiration and intraoperatively. During surgery, a minimum of five samples from different sites were taken during surgery and sent to the laboratory in sterile conditions. All the cultures were studied for aerobic, anaerobic bacteria, and mycobacteria, as well as for fungal growth. At least 21 days of incubation were necessary to declare a PJI as culture-negative. If preoperative and intraoperative culture results disagreed, the microorganism from intraoperative cultures (with ≥ 2 positive samples) was deemed responsible for the PJI. Most of the patients underwent one-stage exchange. In two-stage treatments, cultures were repeated at reimplantation, but our data refer to isolates from the first stage to ensure consistency in our data. Culture-negative PJI cases were excluded from the study. This decision was based on the retrospective nature of the data, which made it impossible to rule out the potential influence of prior antibiotic treatment, improper sample handling, or missing data as causes of the negative culture results.

### Statistical analysis

Quantitative variables were described using metrics such as the number (*n*), mean (average), and standard deviation (SD). The distribution of categorical data is presented using absolute and relative frequencies. To compare the distribution of metric variables in independent groups, the initial step involved conducting the Shapiro–Wilk test to assess the normality of the data. If the assumption of a normal distribution was not disproven (*p* > 0.05), the comparison between two groups proceeded with the *t*-test. In instances where the assumption of normal distribution was rejected, the Mann–Whitney *U* test was utilized for two groups. Fisher's exact test was applied to compare the frequency distributions of a categorical variable across independent groups. For chosen variables exhibiting statistical significance (*p* < 0.05), a binary logistic regression model, incorporating odds ratios (OR) and a 95% confidence interval, was further computed. All tests were conducted as two-sided, and significance was determined with a *p* value < 0.05. The statistical analysis was carried out using the SPSS software program (SPSS, Inc., Chicago, IL, USA).

## Results

A total of 4261 patients were included in the study, with a mean age of 68.0 ± 11.0 years. Most patients were men (56.2%). The mean BMI was 28.71 ± 5.50 kg/m^2^, and the mean Charlson Comorbidity Index was 2.91 ± 2.05. For the two different groups, the CKD group included 409 patients, 54.3% male, mean age 73.83 ± 8.90 years, a higher BMI than the general population (29.88 ± 5.90 kg/m^2^), and a clearly higher CCI of 6.15 ± 2.35. The control group, instead, was made up of 3852 patients, 56.4% male, with a mean age of 67.4 ± 11.1 years and with mean BMI of 28.58 ± 5.44 kg/m^2^. The mean CCI in this group was 2.56 ± 1.68. In patients with CKD, the prevalence of diabetes mellitus was 35.9% versus 10.8% in the control group. Demographic data are summarized in Table 1.

**Table 1:** Demographic data

	CKD group	Control group	Total population	*p*-Value
Patients (*n*)	409	3852	4621	
Age (mean, SD)	73.83 ± 8.90	67.44 ± 11.08	68.01 ± 11.06	< 0.001*
Male ratio (%)	54.3%	56.4%	56.2%	0.408
BMI (mean, SD)	29.88 ± 5.90	28.58 ± 5.44	28.71 ± 5.50	< 0.001*
CCI (mean, SD)	6.15 ± 2.35	2.56 ± 1.68	2.91 ± 2.05	< 0.001*
DM (*n*, %)	147 (35.9)	416 (10.8)	563 (12.2%)	< 0.001*

*SD* standard deviation, *BMI* body mass index, *CCI* Charlson Comorbidity Index, *DM* diabetes mellitus

^*^Statistically significant value (*p* < 0.05)

The comparison in comorbidities rates among the two groups was assessed through a chi-squared test. Fisher's exact test was used if needed. All significant results are summarized in Table 2.

**Table 2:** Patient-related comorbidities and concurrent diseases, comparing CDK group and control group

Comorbidity	Control group, *n* (%)	CKD group, *n* (%)	*p*-Value	OR	95% CI	
Cerebrovascular disease	147 (3.8%)	32 (7.8%)	< 0.001*	2.139	1.439	3.181
Erysipelas	46 (1.2%)	13 (3.2%)	0.001*	2.716	1.455	5.071
Osteomyelitis	204 (5.3%)	33 (8.1%)	0.020*	1.569	1.070	2.301
DVT	175 (4.5%)	33 (8.1%)	0.002*	1.844	1.253	2.715
PE	95 (2.5%)	19 (4.6%)	0.009*	1.927	1.165	3.188
Chronic respiratory diseases	255 (6.6%)	53 (13.0%)	< 0.001*	2.100	1.532	2.878
COPD	156 (4.0%)	39 (9.5%)	< 0.001*	2.497	1.730	3.604
Diabetes	421 (10.9%)	108 (26.4%)	< 0.001*	2.924	2.296	3.725
Heart attack	197 (5.1%)	52 (12.7%)	< 0.001*	2.702	1.954	3.738
Heart failure	178 (4.6%)	78 (19.1%)	< 0.001*	4.864	3.643	6.493
Peripheral arterial disease	65 (1.7%)	26 (6.4%)	< 0.001*	3.955	2.480	6.308
Tumor	451 (11.7%)	76 (18.6%)	< 0.001*	1.721	1.317	2.250
Depression	195 (5.1%)	39 (9.5%)	< 0.001*	1.977	1.379	2.833
Hyperlipidemia	353 (9.2%)	71 (17.4%)	< 0.001*	2.082	1.576	2.750
Hypertension	1.466 (38.1%)	303 (74.1%)	< 0.001*	4.652	3.694	5.859
Hypothyroidism	287 (7.5%)	62 (15.2%)	< 0.001*	2.219	1.651	2.983
Coronary heart disease	274 (7.1%)	95 (23.2%)	< 0.001*	3.951	3.045	5.126
Osteoporosis	168 (4.4%)	47 (11.5%)	< 0.001*	2.847	2.025	4.004
Hypokalemia	114 (3.0%)	35 (8.6%)	< 0.001*	3.069	2.071	4.548
UTI	131 (3.4%)	38 (9.3%)	< 0.001*	2.909	1.996	4.240
Atrial fibrillation	267 (6.9%)	92 (22.5%)	< 0.001*	3.897	2.995	5.071
Ileus	43 (1.1%)	15 (3.7%)	< 0.001*	3.372	1.857	6.125
Sleep apnea syndrome	411 (10.7%)	77 (18.8%)	< 0.001*	1.942	1.485	2.539
Rheumatoid arthritis	73 (1.9%)	19 (4.6%)	< 0.001*	2.522	1.506	4.223
Prior revision	863 (22.4%)	115 (28.1%)	0.009*	1.355	1.078	1.703

Chi-squared test was performed. Significant results emerged in numerous comorbidities. The table reports *p* value and odds ratio (OR, 95% CI)

*OR* odds ratio, *CI* confidence interval, *DVT* deep vein thrombosis, *PE* pulmonary embolism, *UTI* urinary tract infections

^*^Statistically significant (*p* < 0.05)

### Microorganisms and microbiological data

**Fig. 1:**
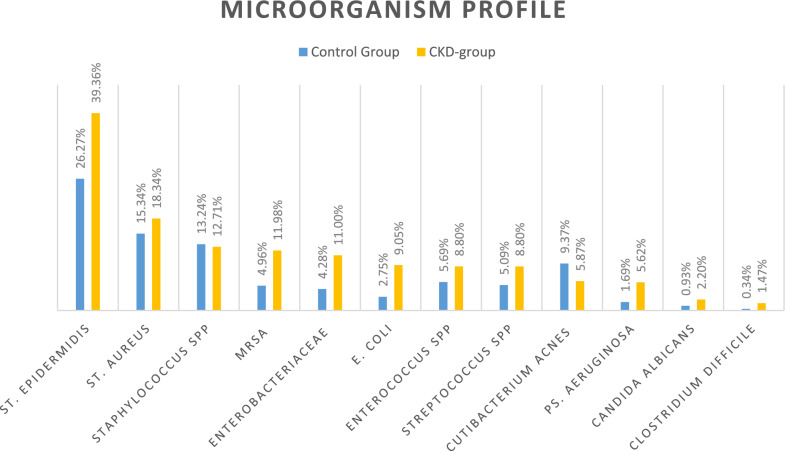
Common bacteria involved in PJI of the hip in CKD group and controls. The group of Enterobacteriaceae includes the following Gram-negative bacteria: *Serratia marcescens*, *Morganella morganii*, *Proteus mirabilis*, *Proteus* spp., *Bacterioides*, *Klebsiella* spp.,*Citrobacter* spp. MRSA, methicillin-resistant *Staphylococcus aureus*

Overall, we identified 70 different species of microorganisms, 52 Gram-positive spp., 28 Gram-negative spp., 3 fungi, and 1 mycobacterium. Polymicrobial infections were 32.59%, being significantly more common in CKD group than controls (47.9% versus 30.9%; *p* < 0.0001). *Staphylococcus* spp. were the most common bacteria in both groups (Fig. 1). The CKD group showed a higher risk of developing infections caused by several microorganisms, in particular *Staphylococcus epidermidis*, *Staphylococcus aureus* (both MSSA and MRSA, *p* = 0.020 and *p* = 0.003, respectively), and Gram-negative Enterobacteriaceae including *Escherichia coli* (*p* < 0.001), *Morganella morganii* (*p* = 0.002), *Serratia marcescens* (*p* = 0.030), and *Klebsiella pneumoniae* (*p* = 0.021). The risk of mycotic infection by *Candida* was significantly higher in the CKD group (*C. albicans* and spp., *p* = 0.035 and *p* = 0.017, respectively). All significant results are summarized in Table 3.

**Table 3:** Microorganism-related differences in PJI of the hip, comparing CDK group and control group

Microorganism(s)	Control group, *n* (%)	CDK group, *n* (%)	*p*-value	OR	95% CI	
*C. albicans*	36 (0.9%)	9 (2.2%)	0.035*	2.385	1.141	4.987
*C. difficile*	13 (0.3%)	6 (1. 5%)	0.007*	4.397	1.662	11.630
*E. faecalis*	178 (4.6%)	30 (7.3%)	0.015*	1.634	1.094	2.440
*M. morganii*	6 (0.2%)	5 (1.2%)	0.002*	7.933	2.410	26.109
*C. acnes*	300 (7.8%)	18 (4.4%)	0.013*	0.545	0.335	0.887
*S. marcescens*	14 (0.4%)	5 (1.2%)	0.030*	3.393	1.216	9.468
*Candida* spp.	80 (2.1%)	16 (3.9%)	0.017*	1.920	1.111	3.316
*K. pneumoniae*	49 (1.3%)	11 (2.7%)	0.021*	2.145	1.106	4.159
*E. coli*	106 (2.8%)	36 (8.8%)	< 0.001*	3.411	2.303	5.052
*P. mirabilis*	42 (1.1%)	15 (3.7%)	< 0.001*	3.454	1.898	6.284
*P. aeruginosa*	65 (1.7%)	23 (5.6%)	< 0.001*	3.472	2.133	5.649
*S. epidermidis*	1012 (26.3%)	161 (39.4%)	< 0.001*	1.822	1.475	2.250
*S. salivarius*	8 (0.2%)	7 (1.7%)	< 0.001*	8.367	3.018	23.193
Poly	1193 (31.0%)	196 (47.9%)	< 0.001*	2.051	1.670	2.519
*Enterococcus* spp.	353 (9.2%)	62 (15.2%)	< 0.001*	1.771	1.323	2.371
MSSA	618 (16.0%)	89 (21.8%)	0.020*	1.455	1.133	1.869
MRSA	55 (1.4%)	12 (2.9%)	0.003*	2.087	1.108	3.930

Chi-squared test and Fisher’s exact test were performed. Significant results emerged in numerous microorganisms causing infections, with increased risk in case group. The table reports *p* values and odds ratio (OR, 95% CI)

*OR* odds ratio, *CI* confidence interval, *MSSA* Methicillin-sensitive *S. aureus*, *MRSA* methicillin-resistant *S. aureus*

^*^Statistically significant (*p* < 0.05)

A logistic regression, adjusted for age, sex, and BMI, was conducted to increase the robustness of the findings and explore potential interdependencies among variables. In this instance, the highest correlation with CKD was confirmed for *Morganella morganii* (*p* = 0.001), *E. coli*, and *Pseudomonas aeruginosa* (*p* < 0.001). PJI caused by *Cutibacterium acnes* was more common in control group than in CKD group (*p* = 0.014). All the results are summarized in Table 4.

**Table 4:** Microorganism-related differences in PJI of the hip, comparing CDK group and control group

Variable(s)	*B*	*p*-Value	Exp(*B*)	95% confidence interval	
*Candida albicans*	0.898	0.043*	2.455	1.028	5.861
*Morganella morganii*	2.315	0.001*	10.121	2.636	38.857
*Cutibacterium acnes*	−0.790	0.014*	0.454	0.242	0.850
*Proteus mirabilis*	0.856	0.017*	2.354	1.164	4.759
*Streptococcus salivarius*	2.213	0.003*	9.144	2.099	39.827
*Escherichia coli*	1.010	< 0.001*	2.745	1.722	4.375
*Pseudomonas aeruginosa*	1.149	< 0.001*	3.153	1.706	5.828
*Staphylococcus epidermidis*	0.584	< 0.001*	1.793	1.389	2.315
Poly	0.587	< 0.001*	1.799	1.398	2.314
MSSA	0.422	0.006	1.525	1.126	2.067

Logistic regression was performed, adjusted for age, sex, and BMI. Significant results emerged in numerous microorganisms causing infection, with increased risk in the case group. The table reports *p* values and relative risk (RR, 95% CI)

*MSSA* methicillin-sensitive *S. aureus*

^*^Statistically significant (*p* < 0.05)

## Discussion

The first important finding of this study is the confirmation that patients in CKD group have a health status different from the general population. Indeed, patients affected by CKD have higher CCI score. Many medical conditions may be responsible for both CKD and PJI. Older age and BMI are well demonstrated as risk factors for PJI [16–19], and in a case–control study comparing PJI with healthy controls, Breznicky et al. found that these comorbidities were present in 24% of patients with PJI compared with 3% in controls [20]. Obesity, DM, and metabolic syndrome are strong risk factors for both PJI and CKD [21]. Chronic renal failure is strongly associated with the full spectrum of cardiovascular diseases, and this situation has been observed not only in end-stage renal disease patients but also in patients with mild or moderate CKD [22, 23]. Even though the aim of the study was not to define the risk of PJI in patients with CKD, on the basis of our results, we presume that the difference in patient characteristics could impact on the microorganism profile.

Regarding the profile of microorganisms, the most common bacteria found in periprosthetic joint infections are Gram-positive bacteria, especially *Staphylococcus* and *Streptococcus* species [24], while fungal PJIs account for less than 1% of all cases [24–26]. This study confirmed the predominance of Gram-positive bacteria, in particular *Staphylococcus* and *Streptococcus* spp., as the main microorganisms responsible for PJIs. However, the relative frequencies demonstrated increased *St. epidermidis* and *St. aureus* and decrease of the other coagulase-negative staphylococci and *Cutibacterium acnes* in CKD group. MRSA was 2.41 times more frequent in CKD that in control group. One reason could be the higher correlation between CKD and previous history of osteomyelitis, as observed in the current study. MSSA is the most frequently identified pathogen across all types of osteomyelitis, followed by *Pseudomonas aeruginosa* and MRSA.

Even though we could not determine how many patients in our study were undergoing hemodialysis at the time of THA, we discussed it as a potential risk factor for different microorganism profiles. Hemodialysis is an important source of hematogenous spread of microorganisms, and dialysis-dependent patients have a much greater risk of MRSA infection [27] in literature. Actually, hemodialysis requires ongoing intravenous access via a central catheter or a dialysis shunt, and numerous studies have linked this greater prevalence to catheter-related infections [27]. In Denmark, the incidence of bloodstream infection was 13.7 per 100 person-years in hemodialysis patients and 0.53 per 100 person-years in a population control [28]. Among the causative organisms of catheter-related bacteriemia isolated in the blood samples, *S. aureus* and MRSA are the most common causative organism. The incidence of *S. aureus* bacteremia in hemodialysis patients was 46.9-fold that of the general population in Denmark [29].

PJI caused by Gram-negative Enterobacteriaceae (including *E. coli*) represented 20% of all PJI in CKD group versus 7% in the control group. *E. coli*, *Klebsiella*, *Serratia marcescens*, and *Morganella morganii* are typically associated to hospital-related infections [30]. They occur more frequently in immunocompromised patients and carriers of invasive medical devices such as catheters. They can cause a wide spectrum of infections such as pneumonia, sepsis, surgical-site infections, and urinary tract infections (UTI). In PJI, their prevalence is about 10% [31]. Patients with CKD are exposed to multiple sources of colonization by nosocomial Gram-negative microorganisms: repeated urinary tract infections [32], higher number of hospitalizations [11], and sepsis [2]. In catheter-related infection in hemodialysis, the most common among the Gram-negative bacteria are *E. coli*, *Enterobacter* species, and *Klebsiella* species [33]. Other studies in Canada found that the relative risks of *Pseudomonas aeruginosa* and anaerobe infections were increased in hemodialysis patients [34, 35].

PJI caused by fungi account for 1% of total PJI, and in 50% of cases *Candida albicans* is the causative microorganism [24–26]. In this study, *Candida albicans* and *Candida* spp. were responsible for 2.3% of hip PJIs in the CKD group. *Candida* spp. are another typical causative microorganism for hospital-related infections, explaining the increased risk in CKD [36].

Another potential factor that may help explain the difference in microorganism profile in patients with CKD could be the difference in symbiotic bacteria present in these patients. Prior research has indicated that CKD is associated with gut dysbiosis, and this relationship is referred to as the “gut–kidney axis” [37, 38]. Recently, gut microbiota dysbiosis has emerged as a significant contributor to the progression of chronic kidney disease (CKD) and its associated complications [39]. Gram-negative Enterobacteriaceae, found to be significantly increased in CKD group, seem to be increased in the gut microbiota of patients with CKD [40]. Stanford et al. demonstrated that individuals with CKD, in contrast to healthy controls, exhibit a decreased number of symbiotic species, coupled with increased numbers of potential pathobionts from the Enterobacteriaceae and Streptococcaceae families [41]. Other findings revealed significant alterations in the diversity of intestinal microbiota in fecal samples between patients with stage 3–4 CKD and healthy subjects. The CKD cohort displayed a higher proportion of Gram-negative bacteria and facultative anaerobes [42]. Chisari, Parivizi et al. found that products from increased gut permeability were significantly increased in hip and knee revision for PJI than in revisions for aseptic loosening [43]. Administration of preoperative oral probiotics needs further investigation, as it may reduce the risk of PJI in patients affected by CKD.

The different profile of microorganisms in patients with CKD might have clinical relevance for treatment outcomes. While the overall success of treatment of hip PJI is 70–90% [44, 45], the increased number of PJI due to MRSA, Gram-negative bacteria, and fungi could increase the risk of treatment failure [31, 46]. Reduced antibiotic susceptibility could decrease the chance of success. A study on urine cultures including *M. morganii*, *S. marcescens*, *Klebsiella*, and others found significant decrease in antibiotic susceptibility [47]. *Klebsiella* spp. and *Enterobacter* spp. can become resistant to majority of beta-lactam antibiotics during treatment. Carbapenem-resistant Enterobacteriaceae such as *E. coli*, *Klebsiella pneumoniae*, and *Enterobacter* species are difficult to treat, as carbapenem resistance is often accompanied by resistance to additional drug classes and can lead to “pandrug”-resistant bacteria [48]. If surgery represents the mainstay of PJI treatment, prolonged antibiotic therapies are still fundamental for success [49]. For example, a study on debridement, antibiotics, irrigation and implant retention (DAIR) showed a good success rate in cases of early acute infection by multidrug-sensitive bacteria, while in the presence of infection by multidrug-resistant bacteria the treatment failure rate was higher [50]. In patients with CKD, we found higher prevalence of infections caused by *Enterococcus* spp., *Streptococcus* spp., and multiple microorganisms that are frequently associated with hospital-acquired colonization or repeated antibiotic treatments. Moreover, a significant proportion of infections were caused by *E. coli*, *Klebsiella*, and other *Enterobacter* species, which often produce extended-spectrum beta-lactamases (ESBLs). These enzymes enable these microorganisms to resist the effects of broad-spectrum cephalosporins and monobactams [51]. In addition, in the Endo-Klinik and Cardiff experiences, PJI caused by those bacteria represent risk factors for failure after one-stage revision [52, 53]. Our hypothesis is that PJI caused by these microorganisms could be associated to antibiotic-resistant phenotypes.

Moreover, owing to renal insufficiency, the use of antibiotics can be restricted in CKD, so that PJI from extremely resistant bacteria can lead to infections that are very difficult to treat [54]. In patients with CKD, two-stage exchange could be indicated for infection control. In selected cases of patients affected by severe comorbidities, 1.5-stage exchange [55] could be a good solution, with implantation of a spacer for indefinite time, which is easier to change in case of infection recurrence or mechanical failure.

There are some limitations to this research. First, it was not possible to determine the complete antibiotic resistance profile of microorganisms; however, it was possible to identify and classify certain strains, such as methicillin-resistant *Staphylococcus aureus* (MRSA) and methicillin-resistant *Staphylococcus epidermidis* (MRSE), because these were explicitly saved in the database under these names. Second, patients in the CKD group were not stratified according to the severity of renal insufficiency. One could expect different microorganism profiles between patients with mild CKD and those who are dialysis-dependent. The different populations characteristics (CCI) can be a confounding factor. However, the strength of the study lies in the large sample size and the complete collection of data regarding microorganism profile.

## Conclusions

Patients affected by CKD are older, more overweight, and affected by a higher number of comorbidities. Renal failure exposes them to PJI caused by microorganisms that might potentially be more drug-resistant and difficult to treat. Knowing in advance the different microorganism profile could help with tailoring an appropriate surgical strategy.

## Data Availability

The datasets used and/or analyzed during the current study are available from the corresponding author on reasonable request.

## Abbreviations

CKD: Chronic kidney disease
PJI: Periprosthetic joint infection
THA: Total hip arthroplasty
CCI: Charlson Comorbidity Index
AVN: Avascular necrosis
UTI: Urinary tract infections
GFR: Glomerular filtration rate
BMI: Body mass index
DM: Diabetes mellitus
MSSA: Methicillin-sensitive *Staphylococcus aureus*
MRSA: Methicillin-resistant *Staphylococcus aureus*
